# Bioavailable turmeric extract for knee osteoarthritis: a randomized, non-inferiority trial versus paracetamol

**DOI:** 10.1186/s13063-021-05053-7

**Published:** 2021-01-30

**Authors:** Shubha Singhal, Nazer Hasan, Kirti Nirmal, Rohit Chawla, Shalini Chawla, Bhupinder Singh Kalra, Anil Dhal

**Affiliations:** 1 Department of Pharmacology, Maulana Azad Medical College, New Delhi, India; 2 Department of Microbiology, Maulana Azad Medical College, New Delhi, India; 3 Department of Orthopedics, Maulana Azad Medical College, Lok Nayak Hospital, Delhi, India

**Keywords:** Knee, Osteoarthritis, Turmeric, Bioavailable, Paracetamol, Pain

## Abstract

**Background:**

To compare the efficacy and safety of bioavailable turmeric extract versus paracetamol in patients with knee osteoarthritis (OA).

**Methods:**

In this randomized, non-inferiority, controlled clinical study, patients of knee OA were randomized to receive bioavailable turmeric extract (BCM-95®) 500 mg capsule two times daily or paracetamol 650 mg tablet three times daily for 6 weeks. The primary outcome measure was Western Ontario and McMaster Universities Osteoarthritis Index (WOMAC) pain subscale. The secondary outcome measures were WOMAC total, WOMAC stiffness, and WOMAC physical function scores. Responder analysis of individual patients at different levels (≥ 20%, ≥ 50%, and ≥ 70%) for WOMAC score was calculated. TNF alpha and CRP levels were evaluated and adverse events (AE) were also recorded.

**Results:**

Seventy-one and seventy-three knee OA patients, respectively in bioavailable turmeric extract and paracetamol groups, completed the study. Non-inferiority (equivalence) test showed that WOMAC scores were equivalent in both the groups (*p* value < 0.05) in all the domains within the equivalence limit defined by effect size (Cohen’s *d*) of 0.5 whereas CRP and TNF-α were better reduced with turmeric extract than paracetamol. After 6 weeks of treatment, WOMAC total score, pain, stiffness, and function scores got a significant improvement of 23.59, 32.09, 28.5, and 20.25% respectively with turmeric extract. In the turmeric extract group, 18% of patients got more than 50% improvement and 3% of patients got more than 70% improvement in WOMAC pain and function/stiffness score and none of the patients in the paracetamol group met the criteria. CRP and TNF-α got significantly reduced (37.21 and 74.81% respectively) in the turmeric extract group. Adverse events reported were mild and comparatively less in the turmeric extract group (5.48%) than in the paracetamol group (12.68%).

**Conclusion:**

The results of the study suggest that bioavailable turmeric extract is as effective as paracetamol in reducing pain and other symptoms of knee osteoarthritis and found to be safe and more effective in reducing CRP and TNF-α.

**Trial registration:**

Clinical Trials Registry – India CTRI/2017/02/007962. Registered on 27 February 2017

**Supplementary Information:**

The online version contains supplementary material available at 10.1186/s13063-021-05053-7.

## Background

Osteoarthritis (OA) is a degenerative disease of the articular joints with progressive nature involving the synovium, articular cartilage, and subchondral bone [1]. It is characterized by the breakdown of cartilage, joint lining, ligaments, and underlying bone [2]. It typically involves an entire joint, with the most commonly affected joints being the hips, knees, hands, and spine. Common manifestations of osteoarthritis are stiffness and pain. There are a variety of risk factors for osteoarthritis, including high-impact sports, obesity, and bone deformities. The prevalence of osteoarthritis increases with obesity and age [3, 4]. Knee OA is the most leading cause of disability and pain in the adult and old age population. An estimated 10 to 15% of all adults aged over 60 have some degree of OA, with prevalence higher among women than men. According to the United Nations, by 2050, people aged over 60 will account for more than 20% of the world’s population. Of that 20%, a conservative estimate of 15% will have symptomatic OA, and one-third of these people will be severely disabled. This means that by 2050, 130 million people will suffer from OA worldwide, of whom 40 million will be severely disabled by the disease (https://www.who.int/medicines/areas/priority_medicines/Ch6_12Osteo.pdf). Knee OA impairs the physical functions and worsens the quality of life [4].

Treatment of osteoarthritis includes a number of pharmacological options. According to the American College of Rheumatology guidelines, paracetamol, also known as acetaminophen (Tylenol), is the first-line therapy for osteoarthritis. Guidelines set forth by the European League Against Rheumatism [5, 6] and the American Pain Society [7] recommend paracetamol (acetaminophen) for arthritis pain (osteoarthritis of the knee, hip, and hand) based on multiple studies testing its safety and efficacy in this patient population [8, 9]. Paracetamol is an effective agent for pain relief due to osteoarthritis as can be seen from a large meta-analysis focused on paracetamol use in osteoarthritis, by far the commonest chronic pain pathology of the elderly [10]. It is effective in reducing pain and improving function in osteoarthritis [11]. If the patient fails acetaminophen, oral and topical non-steroidal anti-inflammatory drugs (NSAIDs) should be preferred, followed by tramadol or intra-articular injections of corticosteroid for additional relief from pain. If patients still have inadequate response to these drugs, opioids are a second-line therapy option for pain relief. There are also evidences that duloxetine can also be used as an adjunct therapy for patients with a partial response to first-line agents [12]. Despite the high prevalence of OA, there is currently no permanent cure or effective treatment that halts or reverses disease progression [13]. While current pharmacologic treatments such as analgesics and NSAIDs provide symptomatic relief, such as relieving pain, they do not exert a clear clinical effect on OA disease prevention or modification. Additionally, in most cases, long-term use of these treatments has been associated with substantial gastrointestinal, renal, and cardiovascular side effects [14]. Thus, there is a clear and urgent need for new therapeutic strategies that are effective and safe for OA treatment.

Dietary supplements, including herbal extracts, have also been examined for the maintenance and treatment of osteoarthritis. A number of dietary supplements (e.g., glucosamine, glucosamine with chondroitin, S-adenosyl-l-methionine, devil’s claw, etc.) have demonstrated efficacy compared to placebo and active controls, while some supplements like methylsulfonylmethane have not [15]. The most important nutritional supplement that has been evaluated and used for treatment of osteoarthritis is curcumin [16]. Curcuminoids are the major phytoconstituents derived from the rhizomes of turmeric (*Curcuma longa*) containing three major components (curcumin, demethoxycurcumin, and bisdemethoxycurcumin). Turmeric has a long history of being used in complementary and alternative medicine and is commonly taken for a variety for health conditions such as arthritis, gastrointestinal complaints, respiratory infections, and even cancer. There is some evidence that shows curcumin has anti-inflammatory, antithrombotic, antioxidant, and antimicrobial activities. The anti-inflammatory effects of curcumin are believed to be a result of inhibiting pro-inflammatory signals such as prostaglandins, leukotrienes, and cyclooxygenase-2. In addition, curcumin has been demonstrated to suppress several pro-inflammatory cytokines and mediators of their release such as tumor necrosis factor-α (TNF-α), IL-1, IL-8, and nitric oxide synthase [17].

Being a highly lipophilic molecule, curcumin suffers from poor absorption and rapid metabolism in vivo, rendering it poorly bioavailable and hence limiting its biological effects. A number of studies support the beneficial effects of curcumin against numerous cancer cell lines and various in vitro tests [18]. It has been found that 10 mg/kg of curcumin given intravenously in rats gave a maximum serum curcumin level of 0.36 μg/mL, whereas a 50-fold higher curcumin dose administered orally gave only 0.06 ± 0.01 μg/mL maximum serum level in rat [19]. Since curcumin is used orally as a nutritional supplement, numerous attempts have been made to improve oral bioavailability through the use of adjuvants like piperine [20] and development of curcumin–lecithin formulation [21], etc. Using piperine to increase bioavailability involves huge risk and close monitoring of patients since it increases the absorption of other drugs/supplements and decreases the hepatic metabolism as well [22]. In a randomized, double-blind, crossover human study, when curcumin was ingested in the form of curcumin-lecithin formulation, only phase-2 metabolites could be detected in the blood [21]. The investigators could not detect free curcumin in the blood.

BCM-95® is one of the bioavailable turmeric formulations comprising of curcuminoids and essential oil of turmeric containing turmerones [23]. Combination of curcuminoids with turmerones (essential oil components of turmeric) has been reported as a powerful tool in the prevention of inflammation and related symptoms [24]. Synergistic effects of curcuminoids with sesquiterpenoids (mainly ar-turmerone) have also been studied by Nishiyama and co-workers for hypoglycemic effects [25]. In a pilot crossover investigation in humans, the relative bioavailability of curcuminoid–essential oil complex was about 6.93-fold higher, compared with normal curcumin, and about 6.3-fold higher, compared with curcumin–lecithin–piperine formula [26]. Interestingly, free curcumin was detected in the blood when it was given as curcuminoid–essential oil complex.

Turmeric extract is used orally as a nutritional supplement for its anti-inflammatory benefits and its low bioavailability is considered a major challenge for its optimal effectiveness. The purpose of this study was to compare the safety and efficacy of bioavailable turmeric extract and paracetamol on patients suffering from knee OA. The primary objective was to assess the improvement in pain with bioavailable turmeric extract and compare with paracetamol. Pain, stiffness, and physical function were measured with Western Ontario and McMaster Universities Osteoarthritis Index (WOMAC) score after 6 weeks of treatment with bioavailable turmeric extract in knee OA patients and compared with paracetamol.

## Methods

### Study design

This was a single-center, randomized, active-controlled, prospective, non-inferiority, intention-to-treat study to compare the efficacy and safety of turmeric extract in pain reduction and functional improvement of knee OA patients with paracetamol. The study was conducted at the Department of Orthopedics, Lok Nayak Jai Prakash Hospital associated with Maulana Azad Medical College, New Delhi, after approval of the Institutional Ethics Committee of Maulana Azad Medical College and Associated Hospitals. No amendments on the accepted protocol were done after starting the study. The clinical trial was prospectively registered in the Clinical Trial Registry of India (CTRI/2017/02/007962). The sample population was recruited from Delhi and the National Capital Region (NCR) of India and adjoining states.

### Study intervention

Generic paracetamol 650 mg and bioavailable turmeric extract were the study interventions used. Turmeric extract (BCM-95®) was provided as 500 mg zero size hard gelatin capsule (Curcugreen® from Arjuna Natural Pvt. Ltd., India). BCM-95® contains a combination of curcuminoids (curcumin, demethoxycurcumin, bisdemethoxycurcumin) with essential oil of turmeric rich in ar-turmerone, which makes it more bioavailable. Each capsule contained curcuminoids and essential oil complex total not less than 95%, curcuminoids not less than 88%, and curcumin not less than 68%. It has been characterized using ultra-performance liquid chromatography (UPLC) and Fourier transform near-infrared (*FT-NIR*) spectrometer (Additional file 1).

Paracetamol is manufactured in huge quantities worldwide. The starting material for the commercial manufacture of paracetamol is phenol, which is nitrated to give a mixture of the *ortho* and *para*-nitrotophenol. The *o*-isomer is removed by steam distillation, and the *p* nitro group reduced to a *p*-amino group. This is then acetylated to give paracetamol [27]. Paracetamol is an effective comparative agent for pain relief due to osteoarthritis as can be seen from a large meta-analysis focused on paracetamol use in osteoarthritis, by far the commonest chronic pain pathology of the elderly [10]. The study doctor assured that none of the participants was on turmeric-based products or NSAIDs.

### Participant selection

The inclusion criteria for recruitment of patients were (1) patients with diagnosis of knee osteoarthritis based on ACR criteria within age group of 40–80 years, (2) patients who have not received any NSAIDs or any other analgesic within 24 h, (3) patients with chronic knee pain (i.e., knee pain at least every other day during the month preceding inclusion), (4) patients with radiologic knee osteoarthritis (Kellgren–Lawrence grades 2–4), and (5) patients capable of comprehending the study instructions.

The exclusion criteria were patients with osteoarthritis linked to metabolic arthropathy, history of recent trauma (< 1 month) responsible for knee pain, knee steroid injection in the previous month, serious comorbid conditions, pregnant or breastfeeding women, and patients who were allergic to paracetamol, ibuprofen, or turmeric.

### Study procedure

The study was carried out in accordance with the principles of the International Conference on Harmonization (ICH) and Good Clinical Practice (GCP) and World Medical Association’s Declaration of Helsinki. Written informed consent was obtained from all subjects by the principal investigator before initiating study-related procedures. Patients with knee osteoarthritis diagnosed by an orthopedic surgeon after clinical and radiographic analysis were selected in the study.

### Sequence generation, allocation concealment, and implementation

Participants were randomly divided into two groups (A and B) using a computer-generated randomization schedule with permuted blocks of random size prepared by the study biometrician. The study medication (turmeric extract or paracetamol) was packaged according to the randomization schedule. Allocation was concealed by using serially numbered, identical, opaque, and sealed containers. Neither the pharmacist nor the biometrician has direct contact with the participants, nor do they have influence in treatment allocation. The study investigators, the research staff dispensing the medication, and the participants will remain blind to the treatment allocation. Study medication was issued by the research staff sequentially to the participants. In the study, patients and physician could not be blinded once randomization and allocation of investigational products has occurred due to the fact that turmeric extract and paracetamol were very different in shape. Patients fulfilling eligibility criteria receive either turmeric extract 1000 mg (one capsule of 500 mg BCM-95®; twice daily) or paracetamol 650 mg thrice a day for 6 weeks.

### Assessment

The patient’s knee symptoms were evaluated by the orthopedic surgeon at day 0 and after 6 weeks according to Western Ontario and Mc Master Universities Osteoarthritis Index (WOMAC). WOMAC scale consists of 24 items divided into 3 subscales: pain (5 items), stiffness (2 items), and physical function (17 items). The test questions were scored on a scale of 0–4, which correspond to none (0), mild (1), moderate (2), severe (3), and extreme (4). The score of each subscale is summed up with a possible score range of 0–20 for pain, 0–8 for stiffness, and 0–68 for physical function. The sum of all three scores gives the value of the total WOMAC score (Additional file 2). Comparison of WOMAC pain score after 6 weeks of treatment between turmeric extract and paracetamol was considered as the primary outcome. Comparison of WOMAC total score, WOMAC function subscale, WOMAC stiffness subscale, and inflammatory markers like CRP and TNF-α of the turmeric extract group with the paracetamol group after 6 weeks of treatment were the secondary outcome. Blood samples were collected on day 0 and day 42 (week 6) for evaluation of CRP and TNF-α which are considered markers for inflammation. Adverse events as reported by the patients were also recorded and compared between the groups. Post-trial care and consultation was provided by the Department of Orthopedics, Lok Nayak Hospital, Delhi.

### Data management

Participants’ data were recorded in paper case record forms (CRFs) and stored in numerical order in a box file at the study site with secured and restricted access. The study coordinator conducted biweekly visits at each of the sites to verify each form for completeness and accuracy. At that point, any missing or inaccurate information was rectified, and the checked completed forms were given for independent data entry personnel. Cross-referencing was done with the paper form to ensure completeness of the query correction. Data was analyzed by an independent statistician who was not involved in the study.

### Access to data

After study completion, paper copies of data were archived in secure storage with restricted access. All data collected were kept strictly confidential. The electronic data were stored in a password-protected server with restricted access. Data transfer was encrypted with all data de-identified. Only members of the research team who need to contact study patients or perform data quality control had access to patient information.

### Dissemination plan

The research team will simplify the study findings and disseminate to the community through local media outlets. The results of the study are planned to publish in international scientific peer-reviewed journals and present at international conferences. The outcomes of the project were disseminated to study patients using non-technical language. The scientific paper getting published will be available for dissemination to study participants.

### Statistical analysis

Sample size was calculated by doing two-sample *t* tests for non-inferiority assuming equal variance using PASS2020 software. Since pain was the primary end point, considering an actual mean difference to detect 0.11 for pain, standard deviation of 4.25, non-inferiority margin of 2.2 with a standardized effect size of 0.5, at one-sided significance level of 0.05, considering a drop out of 25%, and study power of 90%, the estimated sample size is 96 per each arm taking into the condition that WOMAC higher scores are worse [28].

The objective of the study was to determine whether the test product (turmeric extract) has equivalent or non-inferior efficacy to the active control (paracetamol). The maximum clinically acceptable treatment difference defined by effect size (Cohen’s *d*) of 0.5 that is acceptable to establish equivalence or non-inferiority for the primary end point (pain) should be within the margin of 2.2 units in WOMAC pain. Hence, an equivalence/non-inferiority test with the derived non-inferiority margin (NIM) was conducted (Additional file 3 shows the calculation of non-inferiority margin). The simplest and most widely used approach to test non-inferiority/equivalence was the two one-sided test (TOST). Using TOST, equivalence is established at the *α* significance level if the lower limit (LCI) and upper limit (UCI) of (1–2*α*) × 100% confidence interval for mean difference of test product and active control (*μ*_T_ − *μ*_C)_ fall within the lower and upper equivalence limit (LL, UL) of clinically acceptable difference (NIM). Thus, using a 90% confidence interval yields a 0.05 significance level for testing equivalence. On the other hand, if the LCI for *μ*_T_ − *μ*_C_ is greater than LL of the margin of equivalence, then non-inferiority of the test product is concluded. Two hypotheses are tested, whether the treatment difference is below the upper equivalence limit, and above the lower equivalence limit. Two *p* values for each of the one-sided tests are obtained. Higher *p* value is taken into consideration and concluded. If this *p* value is less than alpha, then the research hypothesis (of equivalence) is established.

The primary objective was to assess the treatment difference in WOMAC pain scores between turmeric extract and paracetamol at the end of the study for non-inferiority/equivalence using two one-sided test (TOST). The normal distribution of data was checked by the D’Agostino skewness test. Aspin–Welch unequal–variance *T* test or equal–variance *T* test for equivalence using TOST was used accordingly to compare normally distributed unequal variance or normally distributed equal variance data. For non-normally distributed data, Mann–Whitney *U* or Wilcoxon rank-sum location difference test for equivalence using TOST was used to compare between the two groups. Change in WOMAC scores, CRP, and TNF-α between turmeric extract and paracetamol groups at the end of the study were compared by statistically adjusting baseline covariates (if any) using analysis of covariance (ANCOVA) with 2 groups. The level of statistical significance was set to a *p* value of < 0.05. Subgroup analyses of WOMAC scores were performed to analyze the response of subgroups to the treatment. Responder analysis for the reduction in WOMAC pain alone and WOMAC pain along with function/stiffness score at three different levels of response (≥ 20%, ≥ 50%, and ≥ 70%) of the individual patients over 6 weeks was calculated. Wald *Z* test was used for responder analysis.

## Results

**Fig. 1:**
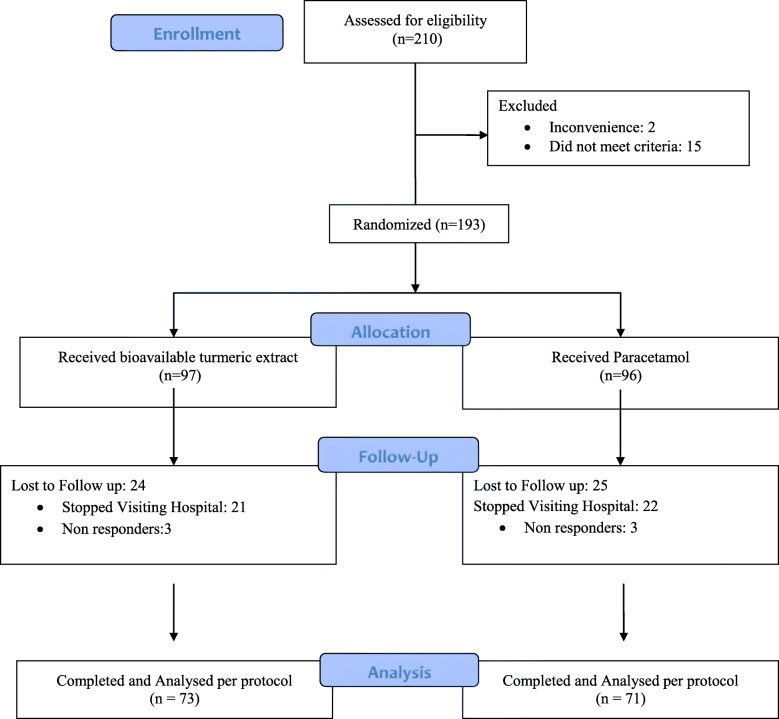
Participant flow diagram

A total of 210 patients were screened and 193 knee OA patients were randomized in the study. Patients with osteoarthritis linked to metabolic arthropathy, knee steroid injection in the previous month, and serious comorbid conditions were excluded from the study. Ninety-seven patients were allocated in the turmeric extract group and 96 patients were allocated in the paracetamol group. Seventy-three patients in the turmeric extract group and 71 patients in the paracetamol group completed the study (Fig. 1). Demographics and baseline characteristics of patients with knee osteoarthritis included in the study analysis are shown in Table 1 (Additional file 4 shows the data of all participants randomized in the study).

**Table 1:** Demographics and baseline characteristics of patients with knee osteoarthritis who completed the study

	Turmeric extract group	Paracetamol group
**Number of participants**	73	71
**Male,** ***n*** **(%)**	20 (27.4)	17 (23.9)
**Female,** ***n*** **(%)**	53 (72.6)	54 (76.1)
**Mean age ± SD (years)**	53.1 (10.9)	50.8 (9.9)
**Mean WOMAC score ± SD**	56.3 (20.5)	50.2(19.5)
**Unilateral knee pain,** ***n*** **(%)**	05 (6.8)	05 (7.0)
**Bilateral knee pain,** ***n*** **(%)**	68 (93.2)	66 (92.9)
**Patients advised knee replacement**	11 (15.1)	12 (16.9)
**Kellergen–Lawrence classification for knee osteoarthritis**		
**Grade II (*n*) (%)**	64 (87.6)	59 (83.1)
**Grade III (*n*) (%)**	09 (12.3)	12 (16.9)

*n* number of participants, *SD* standard deviation

**Fig. 2:**
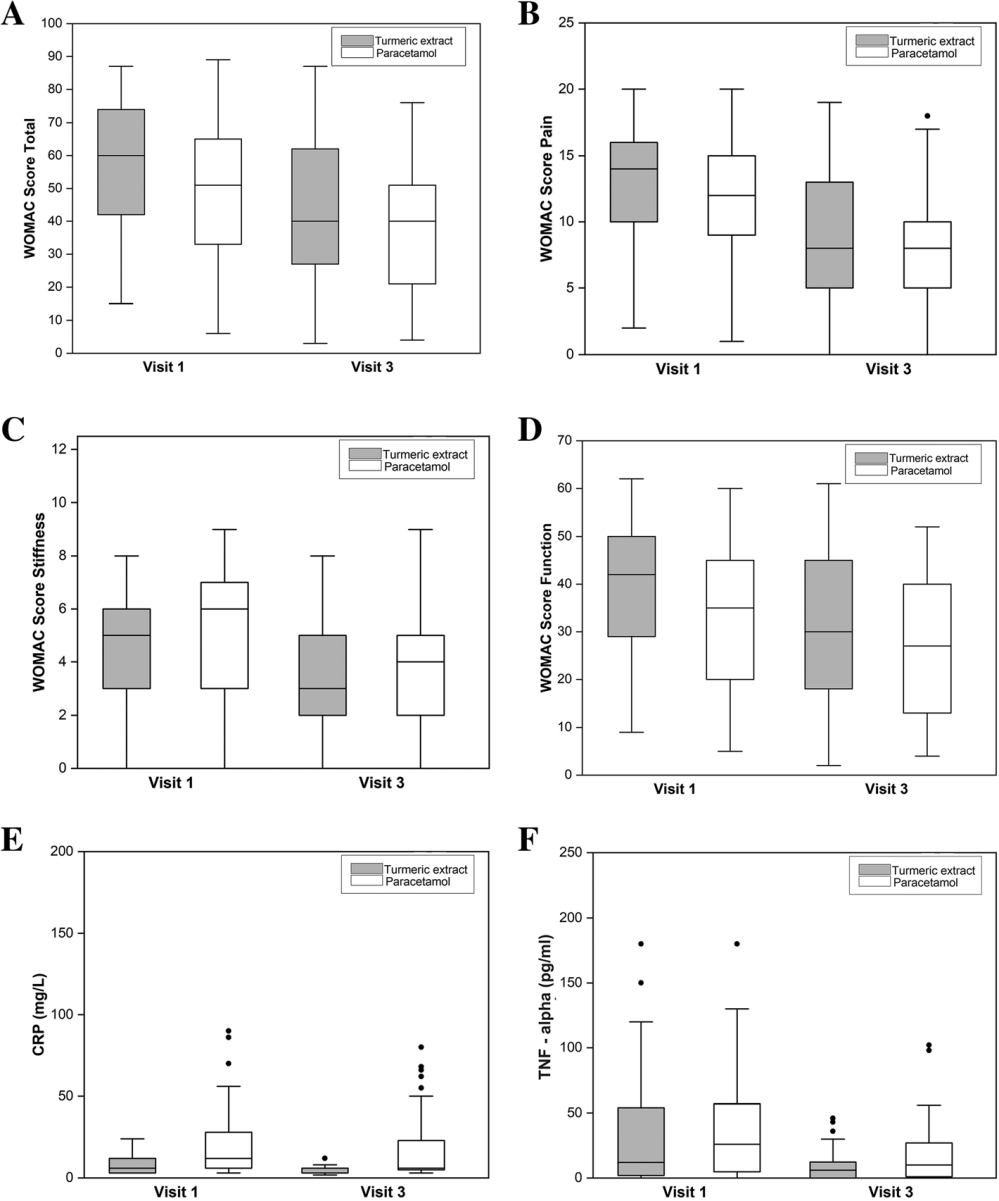
Effect of bioavailable turmeric extract and paracetamol on different domains of WOMAC score, CRP, and TNF-α. **a** WOMAC total score (possible range, 0–96). **b** WOMAC pain score (possible range, 0–20). **c** WOMAC stiffness score (possible range, 0–8). **d** WOMAC function score (possible range, 0–68). **e** CRP—between the groups. **f** TNF-α—within the groups

D’Agostino skewness test showed that WOMAC score data were normally distributed and Aspin–Welch unequal–variance *T* test for equivalence using two one-sided test (TOST) were used for between-group analysis. CRP and TNF-α data were not normally distributed and Mann–Whitney *U* test for equivalence using TOST were used to compare between groups. Non-inferiority (equivalence) test using two one-sided test (TOST) within the equivalence limit defined by effect size (Cohen’s *d*) less than 0.6 showed that WOMAC scores were equivalent in both the groups (*p* value < 0.05) in all domains whereas CRP and TNF-α were non-equivalent in both the groups (CRP *p* value = 0.2589; TNF-α *p* value = 0.0529). The effectiveness of bioavailable turmeric extract in reducing WOMAC score is similar to that of paracetamol and better in reducing CRP and TNF-α than paracetamol (Table 2) (Fig. 2) (Additional file 5 shows the estimated analysis of all randomized subjects).

**Table 2:** Equivalence test of WOMAC scores, CRP, and TNF-α at the end of the study

Parameter	Group (***n***)	Week 6	Median	Percentile		Mean difference ± SE	Confidence level of mean difference		Equivalence test		Effect size^d^
		Mean ± SE		25th	75th		90% LCI	90% UCI	Margin of equivalence^d^	***p*** value	
**Total WOMAC**^b^	Turmeric extract (*n* = 73)	43.01 ± 2.62	40	26	63	5.08 ± 3.39	− 0.54	10.71	LL = −10.8	0	0.526
	Paracetamol (*n* = 71)	37.93 ± 2.16	40	21	51				UL = + 10.8	0.0474^$^	
**Pain**^a,b^	Turmeric extract (*n* = 73)	8.78 ± 0.57	8	5	13	0.87 ± 0.75	− 0.38	2.11	LL = −2.2	0.00004	0.486
	Paracetamol (*n* = 71)	7.92 ± 0.49	8	5	10				UL = + 2.2	0.0389^$^	
**Stiffness**^b^	Turmeric extract (*n* = 73)	3.01 ± 0.24	3	2	5	− 0.59 ± 0.32	− 1.12	− 0.06	LL = − 1.13	0.0481^$^	0.580
	Paracetamol (*n* = 71)	3.61 ± 0.21	4	2	5				UL = + 1.13	0	
**Function**^b^	Turmeric extract (*n* = 73)	31.22 ± 1.90	30	17.5	45	4.81 ± 2.51	0.65	8.97	LL = − 9.00	0	0.589
	Paracetamol (*n* = 71)	26.41 ± 1.64	27	13	40				UL = + 9.00	0.049^$^	
**CRP**^c^	Turmeric extract (*n* = 72)	14.73 ± 8.12	6	3	6				LL = − 3.00	0.2589^$^	0.531
	Paracetamol (*n* = 68)	18.38 ± 2.95	6	4.5	23.5				UL = + 3.00	0	
**TNF-α**^c^	Turmeric extract (*n* = 69)	17.27 ± 4.08	6	0	12.65				LL = − 6.00	0.0529^$^	0.580
	Paracetamol (*n* = 68)	48.65 ± 17.17	10	1	28.5				UL = + 6.00	0	

Margin of equivalence or non-inferiority margin = 0.5 of standard deviation. Effect size = margin of equivalence/standard deviation

*LCI* lower confidence interval of mean difference, *UCI* upper confidence interval of mean difference, *LL* lower limit of margin of equivalence, *UL* upper limit of margin of equivalence, *SE* standard error

^a^WOMAC pain scale is the primary outcome measure

^b^Aspin–Welch unequal–variance *T* test for equivalence using two one-sided test (TOST)

^c^Mann–Whitney *U* or Wilcoxon rank-sum location difference test for equivalence using two one-sided test (TOST)

^d^See Additional file 3 for the derivation of margin of equivalence and effect size

^$^Higher *p* value is considered

To assess the effectiveness of bioavailable turmeric extract, the mean change in efficacy parameters after 6 weeks of treatment was compared with the paracetamol group after baseline covariate adjustment and is represented in Table 3. There was no evidence of a difference in the changes from baseline between the groups for the WOMAC scores and CRP values (*p* > 0.05). The reduction from baseline in TNF-α was statistically significantly greater in the turmeric group compared to the paracetamol group *α* (*p* = 0.0095).

**Table 3:** Comparison of the difference in mean change of efficacy parameters at the end of study between turmeric extract and paracetamol after baseline covariate adjustment

Domain	Group	Mean change ± SE at 6 weeks after baseline covariate adjustment	Mean difference ± SE	*T*	*p* value*
Total WOMAC	Paracetamol (*n* = 71)	40.71 ± 1.07	0.54 ± 1.50	0.359	0.7204
	Turmeric extract (*n* = 73)	40.17 ± 1.05			
Pain	Paracetamol (*n* = 71)	8.35 ± 0.33	− 0.04 ± 0.47	− 0.089	0.9293
	Turmeric extract (*n* = 73)	8.39 ± 0.33			
Stiffness	Paracetamol (*n* = 71)	3.29 ± 0.12	− 0.04 ± 0.17	− 0.22	0.826
	Turmeric extract (*n* = 73)	3.33 ± 0.12			
Function	Paracetamol (*n* = 71)	29.19 ± 0.73	0.87 ± 1.03	0.849	0.3972
	Turmeric extract (*n* = 73)	28.32 ± 0.72			
CRP	Paracetamol (*n* = 68)	18.83 ± 1.77	4.48 ± 2.47	1.81	0.0718
	Turmeric extract (*n* = 72)	14.35 ± 1.72			
TNF-α	Paracetamol (*n* = 68)	46.29 ± 7.75	28.74 ± 10.91	2.63	0.0095
	Turmeric extract (*n* = 69)	17.55 ± 7.69			

*ANCOVA with two groups

Knee OA patients in the paracetamol group and turmeric extract group similarly responded to ≥ 20% reduction in WOMAC pain score and WOMAC pain and function/stiffness score (80% vs 77% and 61% vs 58% respectively). Eighteen percent of knee OA patients in the turmeric extract group got ≥ 50% improvement in WOMAC pain and function/stiffness score and 3% of patients got ≥ 70% improvement. When compared, none of the patients in the paracetamol group got ≥ 50% improvement (18% vs 0%; *p* = 0.0002) (Table 4).

**Table 4:** Responder analysis for WOMAC score at different levels

Responder analysis criteria	Turmeric extract group		Paracetamol group		*p value	Absolute risk difference	Number needed to treat	Relative risk reduction	Relative risk	Odds ratio
	*n* = 73	%	*n* = 71	%						
WOMAC pain score										
≥ 20% reduction	56	77	57	80	0.6024	0.0357	28.02	0.04	0.96	0.81
≥ 50% reduction	21	29	14	20	0.2056	0.0905	11.05	0.46	1.46	1.64
≥ 70% reduction	6	8	3	4	0.99	0.0399	25.04	0.95	1.95	2.03
WOMAC pain and function/stiffness										
≥ 20% reduction	42	58	43	61	0.9756	0.0025	398.69	0	1	0.99
≥ 50% reduction	13	18	0	0	0.0002	0.1781	5.62	126,438	126,439	153,833
≥ 70% reduction	2	3	0	0	0.1602	0.0274	36.5	19,451.1	19,452	20,000

*n* number of participants

*Inequality test of difference of two proportions using the Wald *Z* test (two sided)

### Adverse events

No serious adverse events were reported in both groups. Adverse events observed were mild and self-limiting in character. 12.68% of patients in the paracetamol group reported adverse events which include restlessness (1.41%), abdominal pain/distension (5.63%), dryness of the mouth (2.81%), tingling sensation (1.41%), and melena (1.41%). Only restlessness (4.11%) and tingling sensation (1.37%) were reported for 5.48% of patients in the turmeric extract group.

## Discussion

There is currently no permanent cure for OA or a therapeutic agent with proven evidence to slow or halt the progression of OA [29]. Treatments used to temporarily relieve pain in OA, such as NSAIDs, may also cause severe gastrointestinal, renal, and cardiovascular side effects after long-term use [30]. Apart from the side effects, patients experiencing pain relief without a concurrent improvement in the disease itself may become less conscientious about further protecting the diseased joints (for example by limiting the overuse) and may unknowingly exacerbate the progression of OA. On the other hand, if a drug halts the progression of OA but does not relieve OA-related pain and discomfort, it may not be effective, as patient compliance would likely be very low.

While the pathogenic and etiologic mechanisms for both initiation and progression of OA are not clear, inflammation, over-activated catabolic activity, and oxidative stress responses are considered to be common in both processes [31]. It is also believed that OA is associated with inflammation in articular cartilage, which can cause abnormal joint structure in the knee and hip and it is accompanied with pain. Since the most common treatments (NSAIDs) have serious adverse events in the gastrointestinal tract and cardiovascular system [32], herbal supplements that can mitigate the pain and inflammation have been investigated as potential primary or adjunct therapies for relieving arthritis symptoms.

In earlier studies, the effects of curcumin on attenuating inflammation, formation of reactive oxygen species, and catabolic activity have been suggested in chondrocytes in vitro [33], in human synovial fibroblasts, and in collagen-induced arthritis in mouse models [34]. Furthermore, an anti-inflammatory effect of curcumin on the gene expression of peripheral white blood cells in dogs with OA has also been reported [35]. A number of human clinical trials are available which has reported the efficacy of turmeric extract in the maintenance of OA. In a pilot study on patients with active rheumatoid arthritis, the curcuminoid–essential oil complex was significantly better than diclofenac sodium [36]. Earlier research on the same combination showed better tolerance than diclofenac among patients with knee OA suggesting good alternative treatment option to those who are intolerant to the side effects of NSAIDS [4].

To explore the mechanism behind improved absorption of curcumin from curcumin–essential oil complex, the effects of turmerones on curcumin transport were evaluated in human intestinal epithelial caco-2 cells [37]. The turmerones were found to inhibit p-glycoprotein activities. Results showed that in the presence of turmerones, the amount of curcumin transported into the caco-2 cells in 2 h was significantly increased. The authors suggested the potential use of turmeric extract (including curcumin and turmerones), rather than curcumin alone, for treating diseases. In a very recent study in the dextran sulfate sodium-induced colitis model, anti-inflammatory efficacy, and associated gene expression alterations of curcumin–essential turmeric oil preparation, was investigated in comparison to standard curcumin. The curcumin–essential oil preparation provided superior anti-inflammatory efficacy compared to standard curcumin. In this study, gene expression analysis revealed that anti-inflammatory cytokines including IL-10 and IL-11 as well as FOXP3 were upregulated in the colon by curcumin–essential oil preparation [38]. A few other reports also confirm improved bioavailability of curcuminoids when used in combination with essential oil [39, 40]. The curcuminoid–essential oil complex is extremely safe since LD50 has been reported as > 5000 mg/kg in rats [41].

When individual response of the knee OA patients in WOMAC pain score along with WOMAC stiffness/function score was considered, none of them in paracetamol responded to the treatment but in the turmeric extract group 18% attained ≥ 50% reduction and 3% attained ≥ 70% reduction indicating better response than the paracetamol group. The biomarkers like CRP and TNF-α got significantly reduced as compared to baseline values for both the groups. The most important benefit of turmeric extract is very minimum side effects as compared to NSAIDs. Being a highly bioavailable supplement, low dosage of turmeric extract will be sufficient to get clinical benefits.

## Conclusion

The results clearly indicate that bioavailable turmeric extract is as effective as paracetamol in improving the physical functions and alleviating pain and stiffness of patients suffering from knee OA. CRP and TNF-α were significantly reduced in knee OA patients with bioavailable turmeric extract over a period of 6 weeks and found to be safe.

## Supplementary Information

**Additional file 1.** UPLC chromatogram and FT-NIR graph of bioavailable turmeric extract (BCM-95®)

**Additional file 2.** WOMAC Score

**Additional file 3.** Calculation of Non-inferiority margin

**Additional file 4.** Baseline data of all the randomized subjects with knee osteoarthritis

**Additional file 5.** Estimated analysis of all the randomized subjects

## Data Availability

The datasets used and/or analyzed during the current study are available from the corresponding author on reasonable request.

## Abbreviations

ACR: American College of Rheumatology
AE: Adverse events
CRP: C-reactive protein
IL: Interleukin
NSAIDs: Non-steroidal anti-inflammatory drugs
OA: Osteoarthritis
TNF­α: Tumor necrosis factor-alpha
WOMAC: Western Ontario and McMaster Universities Osteoarthritis Index
