# Supplementary material for: Bioavailable turmeric extract for knee osteoarthritis: a randomized, non-inferiority trial versus paracetamol
Source: Trials. 2021 Jan 30;22:105. doi: 10.1186/s13063-021-05053-7 (PMC7847013; doi:10.1186/s13063-021-05053-7)
Supplement: Supplementary file 1 — Additional file 1. UPLC chromatogram and FT-NIR graph of bioavailable turmeric extract (BCM-95®) [file 13063_2021_5053_MOESM1_ESM.docx]

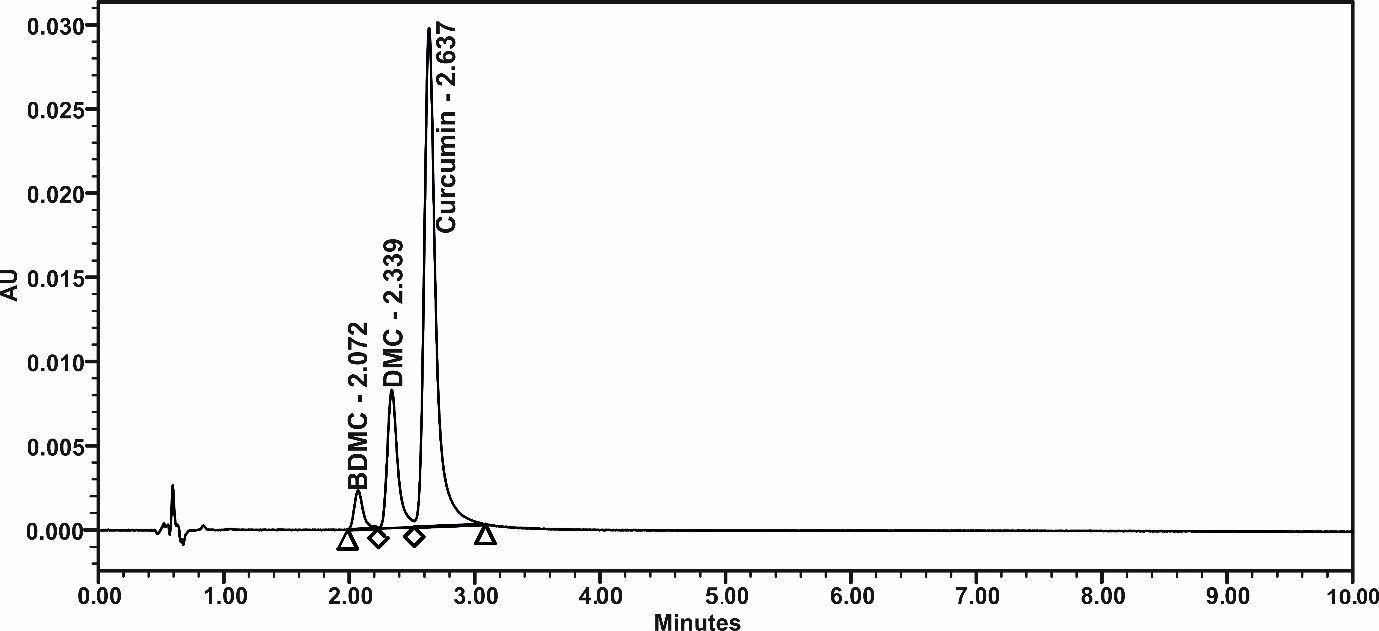


**UPLC chromatogram of bioavailable turmeric extract (BCM-95^®^)**

(curcumin eluted at 2.637 min, demethoxycurcumin (DMC) eluted at 2.339 min and bisdemethoxycurcumin (BDMC) eluted at 2.072 min)


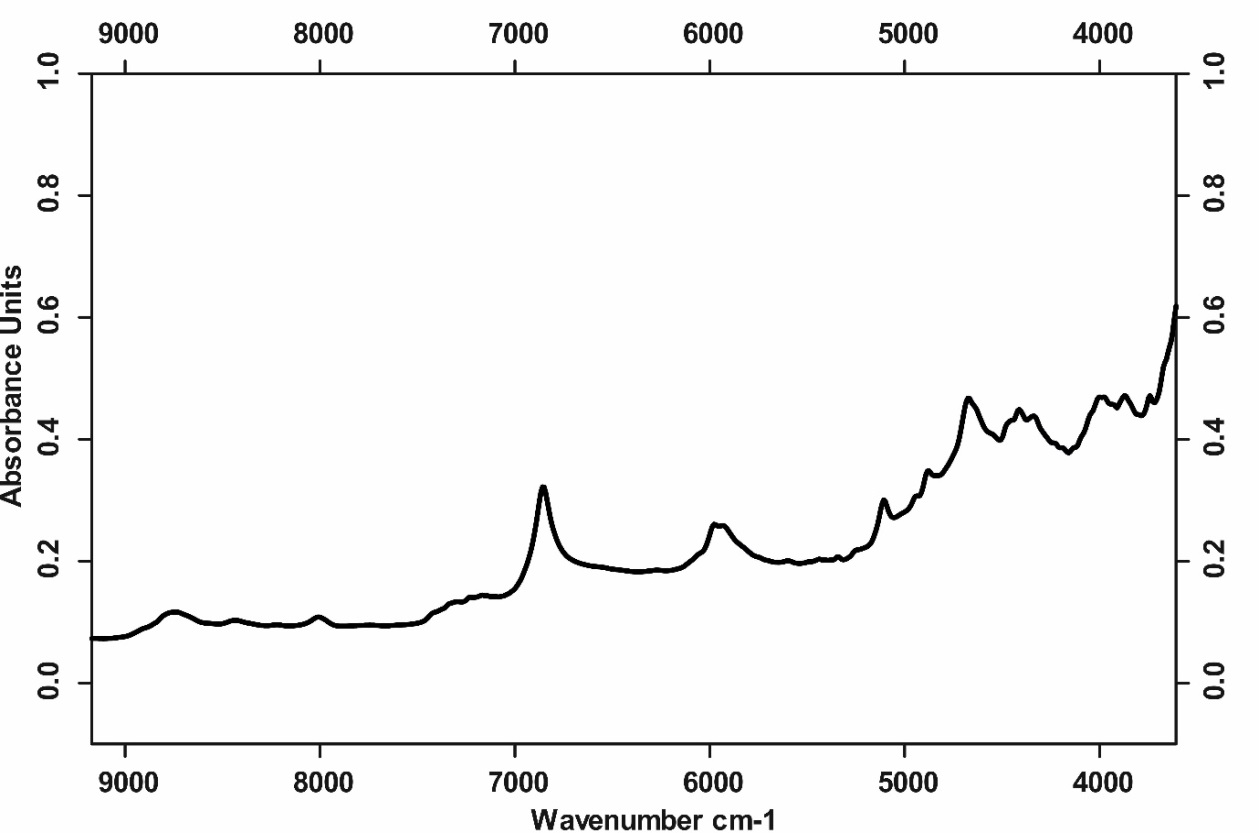


**FT-NIR graph of bioavailable turmeric extract (BCM-95^®^)**
