# Supplementary material for: Bioavailable turmeric extract for knee osteoarthritis: a randomized, non-inferiority trial versus paracetamol
Source: Trials. 2021 Jan 30;22:105. doi: 10.1186/s13063-021-05053-7 (PMC7847013; doi:10.1186/s13063-021-05053-7)
Supplement: Supplementary file 2 — Additional file 2. WOMAC Score [file 13063_2021_5053_MOESM2_ESM.docx]

**The Western Ontario and McMaster Universities Osteoarthritis Index**

**(WOMAC)**

Name:_ _ _ Date: _

Instructions: Please rate the activities in each category according to the following scale of difficulty:

0 = None, 1 = Slight, 2 = Moderate, 3 = Very, 4 = Extremely

Circle **one number** for each activity

| Pain | 1. Walking | 0 | 1 | 2 | 3 | 4 |
| --- | --- | --- | --- | --- | --- | --- |
|  | 2. Stair Climbing | 0 | 1 | 2 | 3 | 4 |
|  | 3. Nocturnal | 0 | 1 | 2 | 3 | 4 |
|  | 4. Rest | 0 | 1 | 2 | 3 | 4 |
|  | 5. Weight bearing | 0 | 1 | 2 | 3 | 4 |
| Stiffness | 1. Morning stiffness | 0 | 1 | 2 | 3 _ | 4 |
|  | _2. Stiffness occurring later in the day | 0 | 1 | 2 | 3 | 4 |
| Physical Function | 1. Descending stairs | 0 | 1 | 2 | 3 | 4 |
|  | 2. Ascending stairs | 0 | 1 | 2 | 3 | 4 |
|  | 3. Rising from sitting | 0 | 1 | 2 | 3 | 4 |
|  | 4. Standing | 0 | 1 | 2 | 3 | 4 |
|  | 5. Bending to floor | 0 | 1 | 2 | 3 | 4 |
|  | 6. Walking on flat surface | 0 | 1 | 2 | 3 | 4 |
|  | 7. Getting in / out of car | 0 | 1 | 2 | 3 | 4 |
|  | 8. Going shopping | 0 | 1 | 2 | 3 | 4 |
|  | 9. Putting on socks | 0 | 1 | 2 | 3 | 4 |
|  | 10. Lying in bed | 0 | 1 | 2 | 3 | 4 |
|  | 11. Taking off socks | 0 | 1 | 2 | 3 | 4 |
|  | 12. Rising from bed | 0 | 1 | 2 | 3 | 4 |
|  | 13. Getting in/out of bath | 0 | 1 | 2 | 3 | 4 |
|  | 14. Sitting | 0 | 1 | 2 | 3 | 4 |
|  | 15. Getting on/off toilet | 0 | 1 | 2 | 3 | 4 |
|  | 16. Heavy domestic duties | 0 | 1 | 2 | 3 | 4 |
|  | 17. Light domestic duties | 0 | 1 | 2 | 3 | 4 |

Total Score: _ _ / 96 = _%

Comments / Interpretation (to be completed by study staff only):
