# Supplementary material for: Bioavailable turmeric extract for knee osteoarthritis: a randomized, non-inferiority trial versus paracetamol
Source: Trials. 2021 Jan 30;22:105. doi: 10.1186/s13063-021-05053-7 (PMC7847013; doi:10.1186/s13063-021-05053-7)
Supplement: Supplementary file 3 — Additional file 3. Calculation of Non-inferiority margin [file 13063_2021_5053_MOESM3_ESM.docx]

**Non-Inferiority Margin Calculation prior to study: Basis of Calculation**

| Calculation of Non-inferiority Margin (WOMAC score) | | | | | | | | | | | | |
| --- | --- | --- | --- | --- | --- | --- | --- | --- | --- | --- | --- | --- |
|  | No of Questions | Score/each question | | Total score | | | Theoretical  Range | Assumed  Range | SD | Non-Inferiority Margin | Effect Size |  |
|  |  | Maximum | Minimum | Theoretical | | Assumed |  |  |  |  |  |  |
|  |  |  |  | Max | Min | Minimum |  |  |  |  |  |  |
| Pain | 5 | 4 | 0 | 20 | 0 | 3 | 20 | 17 | 4.25 | 2.2 | 0.518 |  |
| Stiffness | 2 | 4 | 0 | 8 | 0 | 1 | 8 | 7 | 1.75 | 1.13 | 0.646 |  |
| Function | 17 | 4 | 0 | 68 | 0 | 9 | 68 | 59 | 14.75 | 9 | 0.610 |  |
| Total WOMAC | 24 | 4 | 0 | 96 | 0 | 12 | 96 | 84 | 21 | 10.8 | 0.514 |  |

In the absence of any previous data, we calculated the effect size for total WOMAC score and all its subscales as follows.

1. Calculated the total maximum theoretical score possible after assigning the maximum score for each question. Theoretical minimum total is 0 since each question has the lowest score 0 representing no effect of the respective subscale and total WOMAC score.
2. Calculated the total minimum score after assigning a score of 1 for each question that represents a mild effect for at least 50% or more of the questions for each sub scale.
3. Range (assumed) is calculated by subtracting the total minimum score (assumed) from the total maximum theoretical score.
4. SD is calculated by dividing Range by 4 (Xiang Wan et al 2014 and NCSS Reference manual)

Assume an NIM so that the effect size is 0.5 (approx.) for primary outcome and Total WOMAC and the effect size for stiffness and function to be approx. 0.6

Stiffness and Function (Reason for assuming and high Effect Size)

Stiffness in osteoarthritis usually occurs in the morning, after periods of inactivity or especially in the evening [Bijlsma JWJ et al. 2011]. The stiffness typically resolves within minutes and is relieved by motion of the joint [Altman RD, 2011]. As osteoarthritis progresses only joint motion (function) becomes restricted [Altman RD et al.]. Hence, in order to relate a better feeling in these sub scales to the effect of test material we assumed that we must have an effect size at least 0.6 or above for these two sub scales.

NIM for Bio markers

| Calculation of Non-inferiority Margin (Biomarkers) | | | | | | | | | | |
| --- | --- | --- | --- | --- | --- | --- | --- | --- | --- | --- |
|  | Reported | | | Assumed | |  |  |  |  |  |
|  | Median | 1^st^ quartile | 3^rd^ quartile | 1^st^ quartile | 3^rd^ quartile | IQR (Reported) | IQR assumed | SD | NIM | Effect size |
| CRP (mg/L)) | 2.4 | 1 | 5.1 | 1 | 9 | 4.1 | 8 | 5.9 | 3 | 0.506 |
| TNF -α (pg/mL) |  |  |  | 1 | 16 |  | 15 | 11.1 | 6 | 0.540 |

CRP

In a study (Spector et al 1997) the median, IQR reported are given in the above table. The normal range for CRP is 0-10mg/L ((Leticia A et al). Spector et al (1997) reports that the CRP values can range between 10 and greater than 400. Hence we assumed an IQR of 8 after assigning 3^rd^ quartile CRP value 9. SD is calculated by dividing IQR by 1.35 after assuming the data will be normal. (Xiang Wan, 2014). NIM is fixed so that we get an effect size of about 0.5.

TNF-α

The normal range of TNF-ά is 0-16pg/mL (Leticia A et al). Hence we assumed the 1^st^ quartile and 3^rd^ quartile value to be 1 and 16 respectively so that the IQR is 15. SD is calculated by dividing IQR by 1.35 after assuming the data will be normal. (Xiang Wan, 2014). NIM is fixed so that we get an effect size of about 0.5.

Reference:

1. Altman RD. Osteoarthritis in the elderly population. In: Nakasato Y, Yung RL, eds. *Geriatric Rheumatology: A Comprehensive Approach*. New York, NY: Springer Science+ Business Media, LLC; 2011:187-196.
2. Bijlsma JWJ, Berenbaum F, Lafeber FPJG. Osteoarthritis: an update with relevance for clinical practice. *Lancet.* 2011; 377:2115-2126.
3. Xiang Wan, Wenqian Wang, Jiming Liu and Tiejun Tong. Estimating the sample mean and standard deviation from the sample size, median, range and/or interquartile range, *BMCMedical Research Methodology* 2014, 14:135
4. NCSS2020 reference manual, Chapter135-6
5. T. D. Spector, D. J. Hart, D. Nandra, D. V. Doyle, N. Mackillop,J. R. Gallimore, and M. B. Pepys Low-Level Increases In Serum C-Reactive Protein Are Present In Early Osteoarthritis Of The Knee And Predict Progressive Disease, Arthritis & Rheumatism Vol. 40, No. 4, April 1997, pp 723-7270 1997, American College of Rheumatology
6. Leticia A. Deveza, Changhai Ding, Xingzhong Jin, Xia Wang, Zhaohua Zhu, and David J. Hunter, Laboratory tests In:, Michael Doherty, Johannes Bijlsma, Nigel Arden, David J. Hunter, and Nicola Dalbeth eds. Oxford Textbook of Osteoarthritis and Crystal Arthropathy 3e,UK, Oxford University Press ,2016:193
