# Supplementary material for: Bioavailable turmeric extract for knee osteoarthritis: a randomized, non-inferiority trial versus paracetamol
Source: Trials. 2021 Jan 30;22:105. doi: 10.1186/s13063-021-05053-7 (PMC7847013; doi:10.1186/s13063-021-05053-7)
Supplement: Supplementary file 4 — Additional file 4. Baseline data of all the randomized subjects with knee osteoarthritis [file 13063_2021_5053_MOESM4_ESM.docx]

**Demographics and baseline characteristics of all the randomized subjects with knee osteoarthritis**

|  |  | **Turmeric extract group** | **Paracetamol group** |
| --- | --- | --- | --- |
| **Number of Participants** | Randomized | 97 | 96 |
|  | Completed study | 73 | 71 |
|  | Lost to follow up | 24 | 25 |
| **Male, n (%)** | Randomized | 29 (29.9) | 27(28.1) |
|  | Completed study | 20 (27.4) | 17 (23.9) |
|  | Lost to follow up | 9 (37.5) | 10 (40) |
| **Female, n (%)** | Randomized | 68 (70.1) | 69(71.9) |
|  | Completed study | 53 (72.6) | 54 (76.1) |
|  | Lost to follow up | 15 (62.5) | 15 (60) |
| **Mean Age ± SD (years)** | Randomized | 52.87 ± 10.95 | 51.47 ± 9.47 |
|  | Completed study | 53.11± 10.87 | 50.83 ± 9.92 |
|  | Lost to follow up | 52.13 ± 11.39 | 53.28 ± 7.95 |
| **Mean WOMAC Pain Score ± SE** | Randomized | 12.89 ±0.47 | 11.92 ±0.41 |
|  | Completed study | 12.93 ±0.51 | 11.96 ±0.49 |
|  | Lost to follow up | 12.75 ±1.09 | 11.80 ±0.75 |
| **Mean WOMAC Stiffness Score ± SE** | Randomized | 4.20 ±0.25 | 4.73 ±0.22 |
|  | Completed study | 4.21 ±0.27 | 5.06 ± 0.25 |
|  | Lost to follow up | 4.17 ±0.56 | 3.80 ±0.40 |
| **Mean WOMAC Function Score ± SE** | Randomized | 38.44 ±1.55 | 33.43 ±1.39 |
|  | Completed study | 39.15 ±1.72 | 33.15 ±1.72 |
|  | Lost to follow up | 36.29 ±3.47 | 34.20 ±2.16 |
| **Mean WOMAC Total Score ± SE** | Randomized | 55.53 ±2.18 | 50.18 ±1.89 |
|  | Completed study | 56.29 ±2.40 | 50.17 ±2.32 |
|  | Lost to follow up | 53.21 ±4.98 | 50.20 ±3.14 |
| **Unilateral knee pain, n (%)** | Randomized | 7 ( 7.22) | 6 (6.25 ) |
|  | Completed study | 5 (6.85) | 5 (7.04) |
|  | Lost to follow up | 2 (8.33) | 1(4.0) |
| **Bilateral knee pain, n (%)** | Randomized | 90 ( 92.78) | 90 (93.75) |
|  | Completed study | 68 (93.15) | 66 (92.96) |
|  | Lost to follow up | 22 (91.67) | 24(96.0) |
| **Patients advised knee replacement** | Randomized | 15 (15.46 ) | 15 (15.63 ) |
|  | Completed study | 11 (15.07) | 12 (16.9) |
|  | Lost to follow up | 4 (16.67) | 3 (12.0) |
| **Kellergen- Lawrence classification for knee osteoarthritis** | | | |
| **Grade II, n (%)** | Randomized | 86 (88.66) | 80 (83.33) |
|  | Completed study | 64 (87.67) | 59 (83.1) |
|  | Lost to follow up | 22(91.67) | 21 (84.0) |
| **Grade III, n (%)** | Randomized | 11 (11.34) | 16 (16.67) |
|  | Completed study | 09 (12.33) | 12 (16.9) |
|  | Lost to follow up | 02 (8.33) | 4 (16.0) |
| n = number of participants  SD = Standard Deviation  SE = Standard Error | | | |
