# Supplementary material for: Bioavailable turmeric extract for knee osteoarthritis: a randomized, non-inferiority trial versus paracetamol
Source: Trials. 2021 Jan 30;22:105. doi: 10.1186/s13063-021-05053-7 (PMC7847013; doi:10.1186/s13063-021-05053-7)
Supplement: Supplementary file 5 — Additional file 5. Estimated analysis of all the randomized subjects [file 13063_2021_5053_MOESM5_ESM.docx]

**Estimated analysis of WOMAC scores, CRP and TNF-α** **at the end of the study of the randomized subjects including lost to follow up**

| **Parameter** | **Group (n)** | **Week 6** | **Median** | **Percentile** | | **Mean difference ± SE** | **Confidence level of mean difference** | | **Equivalence test** | |
| --- | --- | --- | --- | --- | --- | --- | --- | --- | --- | --- |
|  |  | **Mean ± SE** |  | **25^th^** | **75^th^** |  | **90% LCI** | **90% UCI** | **Margin of equivalence** | **p value** |
| **Total WOMAC**^#^ | Turmeric extract (n=97) | 39.25 ± 2.03 | 39 | 23.00 | 54.39 |  |  |  | LL = -10.8 | 0.00003 |
|  | Paracetamol (n=96) | 38.02 ± 1.75 | 39.5 | 24.00 | 50.82 |  |  |  | UL = +10.8 | 0.00092^$^ |
| **Pain^@^**^#^ | Turmeric extract (n=97) | 7.94 ± 0.42 | 8 | 4.82 | 11.37 |  |  |  | LL = -2.2 | 0.00007 |
|  | Paracetamol (n=96) | 7.93 ± 0.39 | 8 | 5.00 | 10.00 |  |  |  | UL = +2.2 | 0.00008^$^ |
| **Stiffness**^*^ | Turmeric extract (n=97) | 2.67 ± 0.19 | 2 | 1.18 | 4.00 | -0.70 ± 0.26 | -1.12 | -0.28 | LL = -1.13 | 0.04654^$^ |
|  | Paracetamol (n=96) | 3.37 ± 0.18 | 3.10 | 2.00 | 4.28 |  |  |  | UL = +1.13 | 0 |
| **Function**^#^ | Turmeric extract (n=97) | 28.64 ± 1.51 | 30 | 15.17 | 40.00 |  |  |  | LL = -9.00 | 0 |
|  | Paracetamol (n=96) | 26.67 ± 1.32 | 27.20 | 15.96 | 37.93 |  |  |  | UL= +9.00 | 0.0011^$^ |
| **CRP**^#^ | Turmeric extract (n=96) | 18.14 ± 8.30 | 6 | 3.00 | 6.00 |  |  |  | LL = -3.00 | 0.1067^$^ |
|  | Paracetamol (n=93) | 16.02 ± 2.25 | 6 | 4.86 | 18.85 |  |  |  | UL= +3.00 | 0 |
| **TNF-α**^#^ | Turmeric extract (n=93) | 17.79 ± 3.20 | 10 | 1.5 | 13.9 |  |  |  | LL = -6.00 | 0.00006 |
|  | Paracetamol (n=93) | 48.81 ± 16.55 | 6 | 0 | 20.5 |  |  |  | UL= +6.00 | 0.00026^$^ |
| ^@^WOMAC Pain scale is the primary outcome measure  ^*^Equal-Variance T-Test for Equivalence using TOST (Two One-Sided Tests)  ^#^Mann-Whitney U or Wilcoxon Rank-Sum Location Difference Test for Equivalence using two one sided test (TOST)  ^$^Higher p-value is considered  LCI = Lower Confidence interval of Mean difference  UCI = Upper Confidence interval of Mean difference  LL = lower limit of margin of equivalence  UL = upper limit of margin of equivalence  SE = Standard error | | | | | | | | | | |

End value of the missing patients were estimated using regression imputation method (Multivariate Normal). The results were similar to Table 2 where participants who completed the study were taken for analysis.
